# The complete genome of *Mycoplasmoides cavipharyngis* strain 117C, a close relative of hemotrophic mycoplasma

**DOI:** 10.1128/mra.00556-24

**Published:** 2025-01-30

**Authors:** Janina Kramer, Christina Zübert, Bruno Huettel, Michael Kube, Ludwig E. Hoelzle

**Affiliations:** 1 Department of Livestock Infectiology and Environmental Hygiene, University of Hohenheim 26558 , Stuttgart, Germany; 2 Department of Integrative Infection Biology Crops-Livestock, University of Hohenheim 26558 , Stuttgart, Germany; 3 Max Planck Genome Centre Cologne, Max Planck Institute for Plant Breeding Research 28303 , Cologne, Germany; University of Notre Dame, Notre Dame, Indiana, USA

**Keywords:** complete genome, de novo, *Mycoplasmoides cavipharyngis*

## Abstract

*Mycoplasmoides cavipharyngis* is one of the closest relatives of the highly host-adapted and uncultivable hemotrophic mycoplasma. The complete genome of strain 117C was constructed from long-reads derived from Pacific Biosciences single-molecule, real-time sequencing technology. The genome is organized into one circular, gapless chromosome with a length of 1,034 kb.

## ANNOUNCEMENT

*Mycoplasmoides (synonym:* [*Mycoplasma*]) *cavipharyngis* (1), initially isolated from the nasopharynx of guinea pigs, is characterized as a glucose-fermenting mycoplasma with a 96.5% identity to the 16S rRNA gene of *Mycoplasmoides fastidiosum* (2, 3). Phylogenetic analyses based on 16S rRNA sequences revealed the formation of a distinct cluster for *M. cavipharyngis* and *M. fastidiosum* within the *Mycoplasmoides pneumoniae* group, designated as the *M. fastidiosum* cluster (3). This cluster is described as being most closely related to the hemotrophic mycoplasma (HM) (3–5). HMs are highly specialized, hitherto uncultivable bacteria that cause infectious hemolytic anemia in various mammals worldwide (6). In contrast, *M. fastidiosum* and *M. cavipharyngis* are considered apathogenic species with established *in-vitro* cultivation systems (2, 7). Therefore, genome information on *M. cavipharyngis* and *M. fastidiosum* is crucial for understanding the evolutionary separation of HM from other mycoplasma species and for deciphering new approaches for an *in-vitro* cultivation system.

*M. cavipharyngis* strain 117C [ATCC 43016] was cultivated in SP-4 medium (ATCC Medium 988, [8]) at 37°C under 5% CO_2_. DNA extraction was performed using the Monarch HMW DNA Extraction Kit (NEB, Frankfurt, Germany), followed by DNA quantification with a Qubit Fluorometer (Thermo Fisher Scientific, Darmstadt, Germany). NEB ultra-long reads were obtained through PacBio single-molecule, real-time (SMRT)-sequencing performed at the Max-Planck-Genome-Centre (Cologne, Germany) on a PacBio Sequel II device using SMRTbell Express Template Preparation Kit 2.0, along with Binding Kit 2.0, and Sequel II Sequencing Kit 2.0 (PacBio, CA, USA). The Sequencing resulted in 91,189 reads with an average length of 10,950 nt. Correction, trimming, and assembly of the achieved reads were performed using Canu v2.2 (9) with the pacbio-hifi option and an expected genome size of 1.0 Mb. Of the 91,189 reads, 18,229 corrected-trimmed reads (*N*_50_ of 12,906) were used for assembly, resulting in a single, circular, gapless contig of 1,065,917 bp in length and 186.63-fold coverage.

Sequence overlap was confirmed by BLAST+v.2.12.0 analysis (10) and removed using Artemis v18.2.0 (11). The final assembled genome was automatically annotated using RAST v2.0 (12) with the chromosome start set 21 bp prior to the *dnaA*. Annotation validation and curation were conducted manually in Artemis v18.2.0 (11) using BlastKOALA v3.0 (13), InterProScan v99.0 (14), and BLAST analysis against the non-redundant protein database (https://www.ncbi.nlm.nih.gov/refseq/). Missing non-coding RNA and ribosomal proteins were added using CMsearch v1.1.4 (15) and BLAST analysis. Quality control and completeness of the chromosome were assessed based on BUSCO v5.5.0 analysis (16) with 171 single-copy orthologs of *Mycoplasmatales* spp. (94.8%). All programs and tools used were run with default settings unless otherwise specified.

The circular genome of *M. cavipharyngis* consisted of 1,034,253 bp with a GC content of 25.28%. The rRNA genes were organized in one contiguous operon and a complete set of tRNAs was present. The coding sequences comprised 768 protein-coding genes and 18 pseudogenes.

## Data Availability

Raw reads were allocated to Sequence Read Archive under accession number SRR28961922. A whole-genome project has been submitted to GenBank under BioProject accession number PRJNA1107253. The annotation has been deposited under accession number CP155569.1.
